# Fermentation of Fruit By-Products as a Tool for Nutritional and Environmental Sustainability

**DOI:** 10.3390/foods15030578

**Published:** 2026-02-05

**Authors:** Doheon Kim, Uyory Choe, Young-Jin Park

**Affiliations:** 1 Department of Food and Nutrition, College of Biomedical and Health Science, Konkuk University, 268 Chungwon-daero, Chungju-si 27478, Republic of Korea; doheun98@naver.com (D.K.); foodtech@kku.ac.kr (U.C.); 2 Department of Medicinal Biosciences, Research Institute for Biomedical & Health Science, College of Biomedical and Health Science, Konkuk University, 268 Chungwon-daero, Chungju-si 27478, Republic of Korea

**Keywords:** fruit by-products, fermentation, polyphenols, chemical waste, sustainability, antioxidant, bioactive compounds

## Abstract

Mounting volumes of fruit processing by-products pose an environmental challenge, yet these wastes harbor rich polyphenol reservoirs locked within plant cell walls. Fermentation has emerged as a green biotransformation strategy to unlock these bound antioxidants without the need for chemical solvents, converting waste streams into value-added nutraceutical ingredients. This review summarizes recent advances in fermenting fruit by-products to boost their total polyphenol content (TPC) and antioxidant capacity, illustrating fermentation’s role in both functional enhancement and sustainable waste valorization. Across diverse fruit substrates, microbial fermentation consistently increases TPC and enhances antioxidant activity, demonstrating significant functional enrichment. More importantly, unlike conventional solvent extraction, fermentation-driven valorization reduces chemical waste and allows full incorporation of the biomass into edible products, including bakery products, beverages, and fermented dairy alternatives. This sustainable approach aligns with circular economy principles by turning food waste into functional ingredients, effectively bridging nutritional enhancement with environmental responsibility. Overall, the findings highlight fermentation as an innovative pathway for waste upcycling in the food system, opening new avenues for antioxidant-rich, zero-waste products and their integration into sustainable food ingredient development, while also indicating that the main barrier to industrial translation lies not in functional efficacy but in process compatibility, reproducibility, and scalability under realistic food processing conditions.

## 1. Introduction

The global food processing industry generates a significant volume of by-products and waste, raising critical concerns regarding environmental sustainability, economic efficiency, and resource utilization. According to estimates reported by the World Bank, global solid waste generation reached approximately 2.01 billion tons in 2016 and is projected to increase to about 3.4 billion tons by 2050, with food-related residues accounting for nearly 80% of the total volume [1]. Materials such as fruit pomace, vegetable peels, cereal bran, and seed hulls are commonly discarded despite being rich sources of bioactive compounds and essential nutrients.

This concern is exacerbated by recent shifts in global food consumption patterns. For example, demand for minimally processed and pre-cut fruits and vegetables has increased significantly in recent years, driven by consumer preferences for convenience, time efficiency, and health-oriented diets. According to an industry report, the global processed fruits and vegetables market is expected to grow from USD 417.6 billion in 2026 to USD 620.6 billion in 2035, representing a compound annual growth rate (CAGR) of 4.5% [2]. While these trends cater to modern consumer lifestyles, they also result in an increased generation of plant by-products during pre-processing steps such as washing, peeling, trimming, and cutting.

Fruit by-products discarded during processing have recently emerged as valuable sources of bioactive compounds, particularly polyphenols. A variety of strategies have been employed to valorize by-products. In the food industry, plant by-products have been incorporated into bakery products to reduce waste and enhance dietary fiber and antioxidant content [3]. Beyond bakery applications, by-products have also been utilized in dairy products such as yogurt, meat formulations like sausages, and soups and jams, serving as a source of functional ingredients and improving overall nutritional quality [4].

Direct consumption of fruits or their addition in whole form to food products remains the ideal way to utilize these resources without producing by-products. However, fruits are typically subjected to peeling, deseeding, and trimming prior to processing, resulting in substantial non-edible fractions such as peels, seeds, and pulps. These fractions account for approximately 30–60% of the total fruit mass in mango, about 60% in orange, and about 80% in pineapple [5,6,7]. Not all portions of these food materials are equally palatable or convenient for consumption, leading to inevitable discards such as peels, cores, hulls, or seeds. These by-products are rich in polyphenols; however, they exist in a bound form that is not easily bioavailable in the gastrointestinal tract [8]. While the direct incorporation of fruit by-products into food products offers nutritional and functional benefits, this approach does not fully harness their phytochemical potential.

It is well known that polyphenols exist in two different forms: free and bound in plant tissues. They are closely associated with the plant cell wall matrix, particularly with dietary fibers. Due to their covalent association with structural components like cellulose, pectin, hemicellulose, lignin, and structural proteins, they require proper chemical or enzymatic hydrolysis for effective release and extraction [9]. Through microbial metabolism, fermentation can promote the breakdown of complex cell wall components such as lignocellulose and pectin, thereby facilitating the release of bound polyphenols and other antioxidant compounds [10]. This biotransformation process has been widely associated with increased TPC and enhanced antioxidant activity in various fermented plant matrices, including improvements in both radical scavenging capacity and reducing power, two key mechanisms by which antioxidants counter oxidative stress [11,12].

Fermentation-derived polyphenols have been increasingly investigated for their potential applications in product development. Such investigations carry significant implications: fermentation-based valorization offers a sustainable route to reduce food waste while generating value-added products rich in natural antioxidants. However, most research to date remains confined to laboratory scales and to specific, seasonal feedstocks.

Reflecting these limitations, this review surveys recent advances in the fermentation of fruit by-products, focusing on changes in total polyphenol content and antioxidant capacity. Emphasis is placed on two fundamental antioxidant mechanisms, free radical scavenging activity and reducing power, as key indicators of functional enhancement. By consolidating current evidence, this review illustrates the functional and sustainable potential of fermented by-products, highlights the novelty of this valorization approach, and identifies critical research needs to guide future innovation in food science and waste valorization.

## 2. Pre-Treatment of By-Products

**Figure 1:**
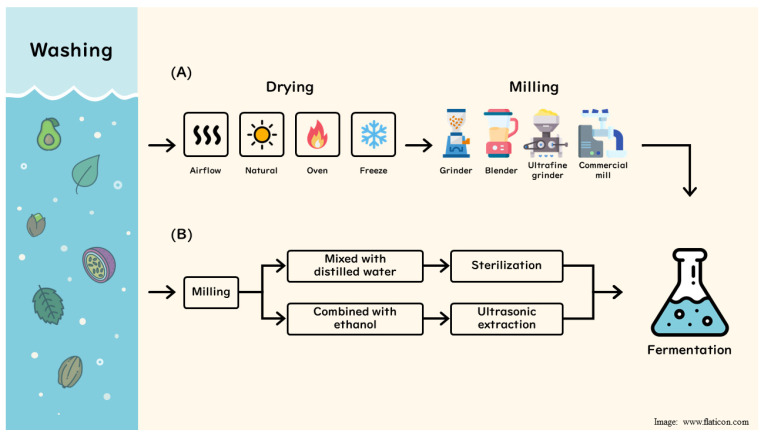
Key pre-treatment methods for fruit by-products prior to fermentation. (**A**) Common pre-treatment route; (**B**) rare pre-treatment routes.

Fruit processing by-products destined for fermentation undergo crucial pre-treatments to ensure a stable, bioactive-rich substrate. Common steps include washing (to remove dirt and inhibitors) and drying the material to prevent spoilage and concentrate phytochemicals, as shown in Figure 1.

Drying methods vary widely from gentle freeze-drying to retain heat-sensitive polyphenols to hot-air/oven drying, airflow dehydration, or natural sun drying, each impacting phenolic retention differently. Once dried, the by-product is typically milled into a fine powder using blenders, standard grinders, ultrafine mills, or commercial grain mills. Finer milling increases surface area and disrupts plant cell matrices, making bound antioxidants more accessible to both extraction and microbes [13].

Beyond standard washing, drying, and milling, specialized routes include rehydration with distilled water followed by pasteurization or autoclaving prior to fermentation and combination with ethanol, followed by ultrasound-assisted extraction prior to fermentation. In summary, a well-designed pretreatment regimen is essential to unlock the full nutraceutical potential of fruit by-products, as it enhances the availability of bioactive compounds for the fermenting microbes and ultimately for human absorption after fermentation.

Following appropriate pre-treatment, fermentation represents a key strategy for the effective utilization of fruit by-products. Unlike direct extraction or simple incorporation into food formulations, fermentation enables the biotransformation of complex plant matrices, promotes the release of bound polyphenols, and improves the stability and functional performance of bioactive compounds [14]. In addition, microbial fermentation has been reported to improve sensory acceptability, support microbial stability, and expand the use of fruit by-products as functional food ingredients [15]. Owing to these combined advantages, fermentation has emerged as a versatile platform for valorizing fruit by-products with diverse compositions and structural characteristics.

Accordingly, the following section summarizes recent studies on individual fruit by-products and discusses how their specific physicochemical properties influence fermentation behavior and functional outcomes.

## 3. Fruit By-Products

In this section, recent studies on the fermentation of fruit by-products are reviewed, with a focus on the fermentation methods and microorganisms employed, as well as the changes in total phenolic content, free radical scavenging activities, and reducing power, as summarized in Table 1.

### 3.1. Acerola

Acerola (*Malpighia emarginata* D.C.) is a nutrient-dense tropical fruit indigenous to Central and South America, particularly cultivated in Brazil, known for its remarkably high vitamin C and bioactive compound content [62]. Acerola’s appealing flavor and exceptionally high vitamin C content have led to its widespread use in products such as juices, jams, and preserves. During the industrial processing of acerola, by-products such as peel, seeds, and residual pulp are generated in significant quantities, accounting for approximately 40% of the total fruit mass [63].

Araújo et al. [16] investigated the fermentation of acerola fruit co-products using *L. paracasei* AL10 and *L. acidophilus* ALA5. After 48 h, ALA5 showed increases in ABTS and FRAP, with ABTS values rising from 20.96 to 28.79 μmol/g and FRAP values increasing from 19.70 to 24.30 μmol FeSO_4_/g. DPPH values in AL10 remained relatively stable, decreasing slightly from 14.19 to 14.10 μmol/g, while ALA5 increased from 8.29 to 9.87 μmol/g but remained lower than AL10. Although ALA5 outperformed AL10 in some assays, the overall changes in antioxidant capacity were limited, suggesting that the functional improvement through fermentation was not substantial.

De Oliveira et al. [17] investigated spontaneous fermentation of acerola by-products and found progressive improvements in TPC and antioxidant activity over 120 h. TPC increased from 779.25 to 1628.30 mg EGA/100 g, while ABTS and FRAP values rose from 1.51 to 1.66 and 361.17 to 428.53 μmol TEAC/100 g, respectively. These results demonstrate that while both probiotic-assisted and spontaneous fermentation enhanced the TPC and FRAP of acerola by-products, the ABTS values differed markedly. In particular, spontaneous fermentation yielded notably lower ABTS (1.51 to 1.66 μmol TEAC/100 g) compared to the probiotic-assisted process (158.00 to 759.00 μmol TEAC/100 g), indicating limited radical scavenging potential under spontaneous conditions.

### 3.2. Apple

Reflecting the well-known adage, “An apple a day keeps the doctor away,” apples (*Malus domestica* Borkh.) are not only perceived as a symbol of daily health but also stand as one of the most widely consumed fruits globally. They are extensively used in the production of juice, beverages, wine, cider, vinegar, and numerous other food products with high commercial value. As a result of such large-scale processing, substantial quantities of by-products collectively known as apple pomace are generated, comprising mainly peel, core, seeds, and residual pulp. It was reported that nearly 25–30% of the apple mass is converted into residues, commonly referred to as apple pomace, during processing, resulting in an estimated 5–7 million tons of apple pomace requiring disposal or valorization annually [64]. Apple pomace contains abundant health-promoting components, including dietary fiber and polyphenols, contributing to its strong antioxidant potential and value as a functional ingredient [65].

Liu et al. [19] fermented apple pomace with *Lactobacillus rhamnosus* L08 to investigate polyphenol biotransformation and antioxidant enhancement. TPC increased from 383.00 to 480.80 μg/mL after 6 days of fermentation. DPPH improved by over 32%, maintaining levels above 90% throughout fermentation. HOSC also increased by an average of 1.14-fold.

Wang et al. [20] conducted solid-state fermentation of apple pomace using a complex probiotic mixture of *Lactiplantibacillus plantarum*, *Saccharomyces cerevisiae*, and *Bacillus subtilis* as an alternative to cellulase treatment. After 6 to 9 days of fermentation, the TPC increased by up to 108.19% compared to unfermented samples.

### 3.3. Araticum

Araticum (*Annona crassiflora* Mart.) is an underutilized fruit native to the Brazilian Cerrado, a biome that covers approximately 25% of the country and is recognized as one of the world’s 25 most biodiverse regions [66]. It is processed into various food products such as juices and jams, while the peel and seeds, which account for 45–55% of the fruit mass [67,68].

de Oliveira et al. [25] reported that fermentation of araticum by-products with probiotic strains significantly enhanced TPC and antioxidant activity. Among the tested strains, *Bifidobacterium animalis* subsp. *lactis* exhibited the highest TPC (657.74 µg EAG/mL) and DPPH activity (2.05 mM ET/mL), along with superior FRAP values (6.67 mM ferrous sulfate/mL) compared to the non-fermented control.

### 3.4. Avocado

Avocado (*Persea americana* Mill.) is a tropical and subtropical fruit native to southern Mexico, now cultivated in various regions worldwide, including Australia, South Africa, and Spain, due to its commercial importance and global demand. Global avocado production has increased rapidly in recent years, supported by strong demand in both fresh fruit markets and processed products such as guacamole and avocado oil [69]. It is also known as “butter pear” for its distinctive shape and creamy texture. An average avocado fruit consists of approximately 20–30% peel and seed generated during processing, leading to an estimated annual production of about 1.2 million tons of by-products [70]. Avocado by-products generated during processing are often discarded without utilization, potentially causing serious environmental problems.

De Montijo-Prieto et al. [26] evaluated the effect of submerged fermentation using various LAB strains on avocado leaf extracts. The highest TPC was observed with *L. plantarum* 748T after 48 h of fermentation, reaching 30.72 mg GAE/g DW. The highest DPPH value was recorded for *P. acidilactici* 5765T after 24 h, at 51.32 mg TE/g DW, while the highest FRAP value was also observed with *L. plantarum* 748T after 48 h, at 96.61 mg TE/g DW. However, overall antioxidant activity after fermentation was generally lower than that of the unfermented control, and the increase in TPC was not consistently significant across strains.

Villasante et al. [27] performed solid-state fermentation (SSF) of avocado seed using *Aspergillus oryzae* and *A. awamori*, resulting in modest but significant increases in TPC and DPPH. After 96 h, *A. awamori* treatment showed 75.79 mg GAE/g TPC and 75.82 μmol TE/g DPPH, compared to 65.49 mg GAE/g and 32.63 μmol TE/g in the unfermented control.

### 3.5. Banana

Banana is a fruit that belongs to the Musaceae family and the *Musa* genus, widely cultivated across tropical and subtropical regions due to its affordability, accessibility, and global consumption. As global demand continues to grow, international banana exports increased from approximately 14.33 million tons in 2000 to about 24.58 million tons in 2021, representing a 1.71-fold increase over two decades [71]. This expansion of the banana market implies a continuous increase in processing activities and associated by-product generation. During processing, banana peels account for approximately 35% of the total fruit weight and are generated as major by-products. In addition, it has been estimated that about 40 million tons of banana residues are produced annually worldwide [72]. These residues are commonly disposed of with limited treatment, potentially contributing to environmental pollution and increasing waste management costs [73]. Moreover, banana by-products are rich in structural carbohydrates and phenolic compounds, making them attractive raw materials for bioconversion and functional ingredient development [74]. Consequently, the sustainable utilization of banana residues is increasingly recognized as a priority for improving resource efficiency and reducing environmental burdens in the fruit processing industry.

Prisacaru et al. [28] investigated the production of banana peel vinegar using alcoholic fermentation with Saccharomyces cerevisiae followed by spontaneous acetic acid fermentation. The resulting vinegars exhibited TPC ranging from 4.03 to 5.72 mg GAE/L and DPPH from 5.42% to 60.92%, depending on processing conditions. Notably, DPPH significantly increased after in vitro intestinal digestion, reaching up to 92.94%.

### 3.6. Baru

Baru (*Dipteryx alata* Vog.) is a native species of the Brazilian Cerrado. Its oval, brown fruits are widely consumed in Brazil’s Midwest and are gaining recognition in international markets, including the United States [75]. The baru fruit consists of 41.9% epicarp and mesocarp, 53.8% woody endocarp, and only 4.3% almond [76]. Despite being rich in dietary fiber and phenolic compounds, baru peel and pulp are indiscriminately discarded during the processing steps aimed at obtaining the edible almond.

de Oliveira et al. [25] investigated the fermentation of baru mesocarp by-products using *Lactobacillus acidophilus* spp. and *Bifidobacterium animalis* subsp. *lactis* Bb-12 in modified MRS medium. After 48 h of fermentation, TPC reached 786.87–982.15 µg EAG/mL, representing a 1.4–1.7-fold enhancement. Antioxidant capacity also improved, with DPPH values of 3.45 to 4.14 mM ET/mL and FRAP ranging from 12.02 to 15.41 mM ferrous sulfate/mL, indicating fermentation-enhanced antioxidant capacity in all treated samples.

### 3.7. Blackcurrant

Blackcurrant (*Ribes nigrum* L.) is predominantly produced in Europe and Asia, which together represent the main production regions worldwide [77]. During industrial processing, pomace can account for approximately 25–30% of the fruit weight, depending on the processing conditions and the type of fruit [78]. Owing to its high phenolic content, particularly anthocyanins, blackcurrant pomace has attracted attention as a source of bioactive compounds with potential antioxidant properties [79].

Sady et al. [29] investigated the antioxidant potential of blackcurrant pomace fermented using three types of microbial processes involving bacteria (B), yeast (D), and a combination of both (BD). After 2 days of fermentation, the bacterial treatment yielded the highest TPC, reaching 78.72 mg GAE/L. Antioxidant capacity measured by ABTS, DPPH, and FRAP assays also showed the highest values in this treatment group. Stalks in 6% salt brine led to a brief rise in phenolic and antioxidant levels, all of which peaked by day 3, suggesting that extended fermentation under such conditions may diminish their functional properties.

### 3.8. Chokeberry

Chokeberry (*Aronia melanocarpa* L.) is native to North America and belongs to the Rosaceae family. It is recognized as one of the richest natural sources of polyphenols, with its high biological activity largely attributed to its abundant phenolic compounds. During juice processing, chokeberry pomace accounts for approximately 16–30% (*w*/*w*) of fruit [80]. Notably, chokeberry pomace contains higher levels of procyanidins than both juice and fresh fruit, underscoring its potential as a valuable source of natural antioxidants [81].

Xiao et al. [30] investigated the effects of solid-state fermentation using *Trichoderma viride* on the polyphenol content and antioxidant properties of chokeberry pomace. The total polyphenol content significantly increased from 67.83 mg/g to 107.21 mg/g, and antioxidant activity measured via DPPH, ABTS, and ORAC assays was markedly enhanced, with DPPH scavenging capacity peaking at day 6.

### 3.9. Granadilla

Granadilla (*Passiflora ligularis* Juss.) is a tropical fruit widely cultivated in highland areas, belonging to the Passifloraceae family, which is well known for its diverse and aromatic passion fruits. Passion fruits are typically processed into juices, during which approximately 50–60% of the total fruit weight, consisting of peel and seeds, is generated as by-products [82].

Santos et al. [31] investigated the effect of solid-state fermentation (SSF) using *Aspergillus niger* on granadilla (*Passiflora ligularis*) seed flour under varying initial moisture contents (50% and 70%) and different extraction solvents (distilled water, 40% or 80% acetone, and 40% or 80% ethanol). Among the tested conditions, the combination of 50% initial moisture and 80% acetone extraction yielded the highest TPC (4713.3 mg GAE/100 g d.b.), representing a 43.6% increase compared to unfermented seeds (3283.0 mg GAE/100 g d.b.). Under the same conditions, antioxidant activity also improved effectively, with ABTS, DPPH, and FRAP values reaching 749.74, 214.99, and 708.02 µmol Trolox/g d.b., respectively.

### 3.10. Grape

Grape (*Vitis vinifera* L.) is one of the most widely consumed fruits worldwide. It is processed into wine, juice, raisins, fresh fruit, and jelly and plays a significant role in the global food industry. According to recent data from the OIV, global fresh grape production reached about 77.7 million tons in 2024, with approximately 30.5 million tons used for wine production and 2.8 million tons for the manufacture of musts and juices [83]. The annual production of grape pomace is estimated at approximately 10.5–13.1 million tons, accounting for about 16.7% (*w*/*v*) of processed grapes, and it is rich in nutritional and bioactive compounds [84]. This by-product retains up to 75% of the fruit’s polyphenols, primarily located in skins and seeds [85]. Given its abundance and functional potential, grape pomace has gained increasing attention as a raw material for value-added applications.

Wang et al. [18] investigated the fermentation of apple and grape pomace mixtures using *Saccharomyces cerevisiae* and *Saccharomyces paradoxus*, producing cider-piquettes. Total phenolic content and antioxidant capacity increased with higher grape pomace ratios, with the grape-only treatment GA1 showing the highest values, with TPC reported as more than 0.55 mg/mL, FRAP 25.97 mM, and DPPH 705.79 µM TEAC/µL, while the apple-only treatment GA2 exhibited the lowest.

Barakat et al. [32] investigated the development of kombucha using grape pomace as a fermentation substrate and employed SCOBY under varying conditions of temperature, sucrose concentration, and fermentation duration. The most effective condition was 20 g sucrose at 20 °C for 7 days, which resulted in the highest TPC at 507.14 mg GAE/L and the strongest antioxidant activity with a DPPH IC_50_ of 1.08 mL/L.

Zhao et al. [33] investigated the effect of solid-state fermentation on grape pomace seeds using four fungal strains. After 12 days of fermentation, the highest TPC was observed with *Eurotium cristatum* FEc.1-1, showing a 9.21-fold increase. The greatest ABTS was achieved with *Monascus anka* GIM 3.592, with a 3.64-fold increase, while the strongest DPPH was recorded with *E. cristatum,* exhibiting a 3.91-fold increase.

Šelo et al. [36] evaluated the effect of solid-state fermentation (SSF) using *Trametes versicolor* on the release of phenolic compounds and the chemical composition of grape pomace using jars and tray bioreactors. Unlike other studies aiming to enhance bioactive profiles, this fermentation led to substantial reductions in total phenolic content and antioxidant activity. After 15 days of SSF, TPC decreased by 76% in jars and 77% in tray bioreactors. Antioxidant activities also declined significantly: DPPH and ABTS values dropped by 82% in jars and by 83% and 72%, respectively, in tray bioreactors, while FRAP values decreased by 77% and 84%. These findings indicate that SSF with *T. versicolor* under these conditions was ineffective in improving the phenolic or antioxidant profile of grape pomace.

Akbulut et al. [37] investigated the effect of replacing black carrot with black grape pomace in shalgam juice production through a two-stage fermentation process using *Saccharomyces cerevisiae*. The TPC increased with higher grape pomace incorporation, reaching the highest level of 1102.47 mg GAE/L in the S5 sample (100% grape pomace) on day 44. The highest DPPH was observed in the S1 sample (100% black carrot) on day 9, with a value of 3.68 mmol TE/L, whereas the maximum ABTS value was 8.63 mmol TE/L in the S5 sample on day 44. Although the TPC increased during fermentation, the differences in antioxidant activities among the shalgam juice samples were not statistically significant.

### 3.11. Guava

Guava (*Psidium guajava* L.) is native to southern Mexico and Central America and is often referred to as the “apple of the poor” due to its low cost, wide availability, and high nutritional value [86]. It is a nutrient-rich tropical fruit known for its antioxidants like lycopene and β-carotene, with growing demand for processed products despite high postharvest losses. A substantial amount of guava by-products is generated during processing, accounting for approximately 30% of the fruit’s total weight [87].

Araújo et al. [16] investigated the fermentation of guava fruit processing co-products using *Lactobacillus acidophilus* LA-05 (GLA5) and *Lacticaseibacillus paracasei* L10 (GL10) to develop synbiotic ingredients. After 48 h of fermentation, GLA5 exhibited consistently higher antioxidant capacity than GL10 across all assays. However, the magnitude of change during fermentation was not substantial. DPPH values remained nearly unchanged, increasing slightly from 15.47 to 15.51 μmol/g. ABTS values showed a modest rise from 23.94 to 28.50 μmol/g, while FRAP values decreased from 27.70 to 19.80 μmol FeSO_4_/g.

### 3.12. Jabuticaba

Jabuticaba (*Myrciaria cauliflora*) is a native Brazilian fruit belonging to the Myrtaceae family, widely consumed in the form of fresh fruit, juice, jelly, and fermented beverages. Despite its short shelf life, jabuticaba has gained attention due to its rich content of anthocyanins, phenolic acids, and flavonoids concentrated primarily in the peel. A substantial amount of waste is generated during jabuticaba processing, and the by-products account for approximately 40% of the whole fruit [88].

Takemura et al. [38] investigated the functional potential of jabuticaba peel flour (JPF) by incorporating it into long-fermented artisanal bread and evaluating changes in antioxidant properties after fermentation and baking. Three bread formulations containing 5%, 7.5%, and 10% JPF (JPF1, JPF2, and JPF3, respectively) were compared with a control. The incorporation of JPF led to marked improvements in oxygen radical absorbance capacity (ORAC), with the most significant increase observed between JPF1 and JPF2. Specifically, ORAC increased from 166.6 to 373.1 μmol TE/g, and further addition to 10% JPF (JPF3) resulted in a modest increase to 434.5 μmol TE/g, suggesting a saturation effect. These findings indicate that jabuticaba peel retains functional stability through fermentation and baking and contributes significantly to the antioxidant capacity of the final product.

### 3.13. Jackfruit

Jackfruit (*Artocarpus heterophyllus* Lam.), a tropical fruit of the Moraceae family, generates a substantial amount of processing waste, estimated to account for approximately 80% of the fruit weight, with an annual by-product generation of about 2.96 million tons [89]. Among these by-products, seed and leaf have been reported to be rich sources of natural bioactive compounds, exhibiting diverse functional properties [90,91].

Bueno-Rojas et al. [39] evaluated the fermentation of kombucha using jackfruit leaves to valorize this underutilized by-product and assess its functional properties. After 10 days of fermentation, the total soluble phenol (TSP) content increased from 0.23 to 0.33 mg GAE/mL. In contrast, antioxidant capacity measured by both ABTS and DPPH assays remained constant at 0.13 mg TE/mL throughout the fermentation period. The FRAP value showed a slight decrease, from 1.69 to 1.62 mg TE/mL. These results suggest that compared to green tea kombucha, the jackfruit leaf kombucha exhibited lower values in TSP and FRAP, while showing comparable ABTS and DPPH.

### 3.14. Lemon

Lemon (*Citrus limon* (L.) Burm.f.) is a citrus fruit belonging to the family Rutaceae and the genus *Citrus*, widely cultivated and consumed for its characteristic acidity, aroma, and nutritional benefits. It is widely processed in the food industry due to its pronounced sourness and rich nutritional content, and by-products such as peel, seeds, and pulp account for about 50% of the fruit [92]. Among these by-products, lemon pomace waste has been identified as a rich source of natural antioxidants, including polyphenols and flavonoids.

Hsu et al. [41] investigated the effect of different fermentation strategies on lemon peel juice (FLPJ), comparing unfermented FLPJ (un-FLPJ), sugar-added fermented FLPJ (SA-FLPJ), and sugar-free fermented FLPJ (SF-FLPJ). Among the three, SF-FLPJ exhibited the highest TPC (1292.58 µg/g) and the lowest EC50 value in the DPPH assay (19,117.66 ppm), indicating superior antioxidant activity. In contrast, SA-FLPJ had reduced polyphenol levels and antioxidant capacity, likely due to the influence of added sugars, while un-FLPJ showed the lowest bioactive enhancement.

### 3.15. Litchi

Litchi (*Litchi chinensis* Sonn.) is a tropical and subtropical fruit belonging to the family Sapindaceae, widely cultivated for its sweet flavor and popularity as a fresh fruit. Although it is often regarded as a superfood owing to its rich nutritional profile, it contributes significantly to global fruit waste because of the large proportion of inedible peel and seed. The peel and seed of litchi account for approximately 30–40% of the total fruit weight, and although the by-products generated during processing are rich in nutrients and bioactive compounds, they are largely discarded as waste [93,94].

Zhang et al. [42] investigated the use of litchi (*Litchi chinensis* Sonn.) seed starch as a fermentation substrate for vinegar production and found that the TPC and DPPH varied significantly depending on the hydrolysis and fermentation method. Among the treatments, the enzymatically hydrolyzed and vinegar-fermented sample (EHS) exhibited the highest TPC at 700.81 mg GAE/L and DPPH at 75.92%, surpassing all acid hydrolysis-based treatments.

### 3.16. Mandarin

Mandarin (*Citrus reticulata* Blanco) is a widely cultivated citrus fruit belonging to the family Rutaceae and the genus *Citrus*. Among citrus varieties, mandarins represent approximately 22% of global production, ranking second in cultivation volume after oranges [95]. During industrial processing, particularly for juice extraction, peels constitute the main by-product, accounting for nearly 30% of the fruit mass [96].

Mamy et al. [43] conducted a study to enhance the antioxidant potential of mandarin peel through solid-state fermentation using *Aspergillus niger*. By optimizing the fermentation medium with the Box–Behnken design, they significantly improved the antioxidant profile of Citrus reticulata peel powder (CRPP). Specifically, TPC increased from 13.73 mg GAE/g in the unfermented control (UF-CRPP) to 17.19 mg GAE/g in the optimized fermented sample (FO-CRPP), while ABTS and DPPH radical scavenging activities improved from 23.65 µmol TE/g and 16.58 µmol TE/g to 34.48 µmol TE/g and 26.08 µmol TE/g, respectively. In all antioxidant indicators, FO-CRPP showed higher values than FI-CRPP, indicating that optimization of the medium further enhanced the bioactive potential.

### 3.17. Mango

Mango (*Mangifera indica* L.) is a tropical fruit belonging to the family Anacardiaceae, and it is the second most traded tropical fruit worldwide [97]. Owing to its rich nutritional profile, it enjoys great popularity in tropical regions and is often referred to as the “king of fruits” [98]. Global mango production has increased substantially, rising from approximately 25 million tons in 2000 to over 57 million tons in 2021 [99]. Mango processing results in the generation of peel and seed waste, which accounts for approximately 30–60% of the fruit weight, with an estimated annual production of about 20 million tons of mango by-products [5]. In the context of ongoing climate change, rising global temperatures may expand the geographic range of climatically suitable habitats for mango cultivation [100]. This expansion could further support increases in mango production in the coming decades, which would in turn lead to a substantial rise in the volume of mango processing by-products generated worldwide.

Vilas-Franquesa et al. [44] evaluated the functional valorization of mango peels through Viscozyme-assisted enzymatic pretreatment followed by fermentation with *Lactiplantibacillus plantarum* (LP01) and *Bifidobacterium animalis* (B501). The combination of VI + B501 showed the greatest improvement in antioxidant properties, with FRAP and DPPH reaching 1.220 mM TE and 3.270 mM TE, respectively. Meanwhile, the highest TPC was observed in the VI + LP01 combination, reaching 3.276 mg GAE/100 mg DM.

Vilas-Franquesa et al. [45] investigated the upcycling of mango seed kernel (MSK) into a functional ingredient via solid-state fermentation (SSF) using *Aspergillus oryzae* and *A. awamori*. Fermentation with *Aspergillus awamori* significantly enhanced the antioxidant potential of mango seed kernel flour. After 96 h, TPC increased to 2.635 mg GAE/g compared to the control (2.402 mg GAE/g), while ABTS and DPPH increased to 0.470 mg TE/g and 14.081 mM TE/g, respectively, surpassing both the unfermented control and the *A. oryzae*-treated samples.

### 3.18. Mulberry

Mulberry (*Morus alba* L.) is a widely cultivated fruit belonging to the family Moraceae, known for its nutritional richness and medicinal benefits. Mulberry is also widely used in the food industry to produce various value-added products such as jam, jelly, wine, syrup, and vinegar, owing to its high sugar content and pleasant flavor. This processing generates mulberry pomace, which accounts for approximately 40% of the total fruit weight [101].

Tang et al. [46] investigated the functional valorization of mulberry pomace through fermentation with *Lactobacillus plantarum*, aiming to enhance its antioxidant capacity and modulate gut microbiota composition. Their study demonstrated that fermented mulberry pomace (FMP) showed modest but statistically significant increases in antioxidant activity compared to non-fermented samples (NFMP), with peak values observed after three days of fermentation. Specifically, the FMP-3d sample achieved the highest DPPH, ABTS, FRAP, and ORAC activities, reaching 275.06, 480.22, 334.19, and 331.05 mg Trolox/100 mL, respectively.

### 3.19. Orange

Orange (*Citrus sinensis*) is a widely consumed citrus fruit belonging to the family Rutaceae and is commonly processed into various food products, including juice, jam, and jellies [102]. It is also regarded as one of the principal dietary sources of vitamin C and citrus flavanones, compounds that largely define its characteristic freshness and nutritional value [103]. During the processing of oranges, approximately 60% of the fruit is converted into by-products such as peel, seed, and pulp, and the annual generation of orange residues is estimated to reach about 55 million tons worldwide [6].

Ganiyu et al. [48] evaluated the functional enhancement of orange peels through solid-substrate fermentation using *Penicillium camemberti*. Compared to unfermented samples, the fermented peels exhibited significantly higher antioxidant potential, with TPC increasing from 0.54 to 2.06 mg GAE/g, DPPH from 60.39% to 81.68%, and FRAP from 9.58 to 10.77 mg AAE/100 g.

Hu et al. [51] investigated the enhancement of citrus pomace functionality through solid-state fermentation with autochthonous probiotics, *Lactobacillus plantarum* (P10, M14) and *Bacillus subtilis* (BF2). Co-fermentation with *B. subtilis* BF2 and *L. plantarum* P10 (BPF) led to a 133.15% increase in TPC and markedly improved antioxidant activity, with DPPH and ABTS rising by 397.8% and 226.1%, respectively.

Recently, Dikmetas et al. [52] investigated the production of functional orange juice using lactic acid bacteria (*Lactobacillus acidophilus*, *L. casei*, and *L. plantarum*) to ferment orange pomace (OP) and evaluated the antioxidant activity and phenolic composition of the resulting products. Among the tested conditions, fermentation with *L. plantarum* for 3 days (FOP-LP, day 3) yielded the highest TPC at 540.67 mg GAE/100 g DW. The strongest DPPH was observed in *L. acidophilus*-fermented pomace (FOP-LA, day 3), reaching 300.98 mg TE/100 g DW, while the highest CUPRAC value was also recorded for the same treatment at 423.01 mg TE/100 g DW.

### 3.20. Pequi

Pequi (*Caryocar brasiliense* Camb.), a native fruit of the *Caryocar* genus, takes its name from an indigenous term meaning “thorny shell” and is traditionally consumed by local populations in Brazil’s Cerrado and Amazon regions. It is mainly used in culinary preparations and in the production of ice cream and liqueurs, while only about 9% of the whole fruit is edible and approximately 91% of its total weight is discarded during processing [104]. Pequi peel contains notably higher levels of phenolic compounds compared to other parts of the fruit, including the pulp [105]. This highlights the potential for valorizing pequi by-products as sources of bioactive compounds.

de Oliveira et al. [25] evaluated the fermentation of pequi by-products using various probiotic strains to enhance their functional properties. Among the tested strains, *Bifidobacterium animalis* subsp. *lactis* Bb-12 exhibited the most effective results in fermenting the pequi matrix, achieving the highest TPC (1472.73 μg EAG/mL) and FRAP (34.54 mM ferrous sulfate/mL). However, fermentation did not improve DPPH, as the highest DPPH value (13.4 mM ET/mL) was observed in the unfermented control.

### 3.21. Pineapple

Pineapple (*Ananas comosus*), a tropical fruit of major economic importance, is extensively cultivated and processed worldwide for fresh consumption, juice production, and canned goods. It ranks as the third most consumed fruit in the world, following bananas and citrus fruits, with an annual production approaching 28 million tons [106]. As a result of large-scale industrial processing, substantial quantities of by-products, primarily peel, crown and core, are generated and account for approximately 80% of the total fruit weight of pineapple [7]. Owing to their richness in phenolic compounds, minerals, and vitamins, pineapple processing residues are increasingly recognized as valuable sources of bioactive and nutritional components. These characteristics make pineapple by-products a promising target for functional valorization, particularly through bioprocessing strategies such as fermentation.

Casas-Rodríguez et al. [53] evaluated the impact of solid-state fermentation (SSF) using *Aspergillus niger* strains on the antioxidant potential of pineapple peel waste. Among the tested strains, *A. niger* HT3 and *A. niger* Aa20 exhibited the most significant improvements. Specifically, the highest DPPH was observed in extracts fermented with *A. niger* HT3, reaching 60.28%, a 1.5-fold increase over the unfermented control. In terms of ABTS, fermentation with *A. niger* Aa20 resulted in the highest inhibition rate of 81.41%, reflecting a 2.8-fold enhancement. Regarding FRAP, *A. niger* HT3 fermentation yielded the highest value at 176.64 mEq Trolox/g, a 2.9-fold increase. These findings highlight the effectiveness of specific *A. niger* strains in enhancing the antioxidant profile of pineapple peel through SSF.

Ortega-Hernández et al. [7] investigated the potential of pineapple peel as a substrate for solid-state fermentation (SSF) using *Lactiplantibacillus plantarum*, *Lacticaseibacillus rhamnosus*, and *Aspergillus oryzae*. Among the tested strains, *L. plantarum* fermentation for five days yielded the highest increase in TPC, reaching a 248.11% improvement compared to unfermented samples. In terms of antioxidant activity assessed via DPPH assay at 1000 µg/mL concentration, *L. plantarum* and *L. rhamnosus* achieved the highest scavenging activity by day 5, with values of 57.51% and 56.02%, respectively.

Lou et al. [55] investigated the antioxidant-enhancing potential of polyphenol-rich pineapple peel powder (PPP) and pineapple dietary fiber (PPF), with or without ultrasound pretreatment, in yogurt fermentation using *Lactobacillus bulgaricus* and *Streptococcus thermophilus*. Among the experimental groups, the yogurt supplemented with ultrasonicated pineapple dietary fiber (NPFU) exhibited the highest TPC, reaching 104.93 µg/mL on day 14, compared to the control group (NN) at 52.21 µg/mL. Similarly, the NPFU group showed the strongest DPPH and ABTS, as evidenced by the lowest IC_50_ values at the end of storage. In line with these findings, the highest FRAP value was also observed in the NPFU group (2.87 mmol/L), indicating enhanced reducing power.

### 3.22. Pomegranate

Pomegranate (*Punica granatum* L.) is a deciduous fruit tree belonging to the family Punicaceae and is widely cultivated in temperate and subtropical regions across the globe. It is primarily processed into juice, although ready-to-eat arils, jams, jellies, and syrups are also increasingly developed as value-added products. Following juice extraction, a substantial portion of the pomegranate, consisting of the rind and seeds, which together make up approximately 54% of the whole fruit, is discarded as processing by-products [107].

Vafajoo et al. [56] employed a biomimetic fermentation approach using *Aspergillus tubingensis* to enhance the antioxidant properties of pomegranate peel. This method does not require added inoculum, toxic solvents, or synthetic enzymes and instead relies on natural fungal colonization and the use of inherent enzymatic activity. As a result, it offers a cost-effective and environmentally benign alternative to conventional extraction techniques. At a 1:10 solid-to-liquid ratio, fermentation for 48 h increased the TPC from 167.7 to 300.3 mg GAE/g DM. The DPPH radical scavenging activity, expressed as gallic acid equivalent (GAE), also increased from 156.44 to 256.34 mg GAE/g DM, confirming the efficacy of this eco-friendly extraction method.

### 3.23. Pomelo

Pomelo (*Citrus grandis*) belongs to the genus *Citrus* and is widely grown in tropical and subtropical areas of Southeast Asia. The peel of pomelo, commonly treated as an agricultural by-product, is a rich source of bioactive compounds with potential applications in food and functional ingredients. Accounting for approximately 30–50% of the total fruit weight, the peel is prone to rapid spoilage and, if not properly managed, may pose an environmental concern [108].

Zheng et al. [57] investigated the effect of solid-state fermentation using *Aspergillus oryzae* on the antioxidant properties of pomelo peel. TPC significantly increased throughout the 12-day fermentation period, reaching a peak value of 74.67 mg GAE/g on day 8, which was approximately 3.8-fold higher than the unfermented control (19.46 mg GAE/g). In parallel, the antioxidant activities measured by DPPH, ABTS, HOSC, and FRAP assays also reached maximum levels on day 8, with values of 3.84 mg VCE/g, 35.49 mg VCE/g, 103.88 mg VCE/g, and 7.03 mg TE/g, respectively. These findings suggest that *A. oryzae*-mediated fermentation effectively enhances the antioxidant potential of pomelo peel.

### 3.24. Rambutan

Rambutan (*Nephelium lappaceum* L.) is a tropical fruit-bearing tree of the Sapindaceae family, widely cultivated in Southeast Asia and other humid tropical regions. Various rambutan-derived products, including jams, jellies, and preserved fruits, are commercially produced, and their processing results in substantial amounts of by-products such as peel, seed, and embryo. These non-edible portions, such as peel, seed, and embryo, together account for approximately 61.3% of the total dry weight of the fruit, highlighting the importance of their utilization in value-added processing [109].

De La Rosa-Esteban et al. [58] explored the solid-state fermentation of rambutan peel using *Saccharomyces cerevisiae* and *Yarrowia lipolytica* under varying conditions. Among the 15 treatments, the highest TPC was 103.66 mg/g in treatment 15. The highest DPPH was 63% in treatment 3, while the ABTS reached 100.00% in treatment 6.

Cerda-Cejudo et al. [59] evaluated the functional valorization potential of fermented Mexican rambutan peel using solid-state fermentation with *Aspergillus niger*. Under conditions optimized through central composite design, the highest TPC reached 73.18 mg/g. Antioxidant capacity was also enhanced, with ABTS and DPPH of 98.44% and 74.73%, respectively.

### 3.25. Raspberry

Raspberry (*Rubus idaeus* L.) is a perennial shrub in the Rosaceae family and ranks third in global small berry market value, after strawberry and blueberry [110]. In industrial applications, raspberries are commonly processed into juice, during which a substantial amount of pomace is generated as a by-product. It has been reported that this pomace represents approximately 9% of the fresh weight of the processed fruit [111]. Raspberry pomace is rich in polyphenols and dietary fiber, and is therefore considered a promising source of bioactive compounds for potential functional food applications.

Sady et al. [29] investigated the impact of microbial fermentation on the functional properties of raspberry pomace, utilizing three fermentation approaches: lactic acid bacteria (B), yeast (D), and a mixed culture of both (BD). Among these, fermentation with *Saccharomyces cerevisiae* (D) for 48 h resulted in the highest TPC of 82.06 mg GA/L. However, the increase was not statistically significant. Fermentation using lactic acid bacteria (B) and mixed cultures (BD) led to reductions in TPC values. In terms of antioxidant activity, yeast fermentation (D) enhanced the scavenging capacity, as measured by ABTS (0.70 mmol Trolox/L), DPPH (0.36 mmol Trolox/L), and FRAP (0.47 mmol FeSO_4_/L).

Stamenković Stojanović et al. [60] investigated the effect of raspberry pomace supplementation and cold storage on the antioxidant properties and polyphenol content of kefir. The addition of raspberry pomace led to a marked increase in both antioxidant activity and TPC throughout the 14-day maturation period. Among the tested samples, the highest TPC (78.24 mg GAE/L) and strongest DPPH (95.91%) were recorded on day 10 in K2, which contained 20% raspberry pomace retained throughout the storage period. These results demonstrate that the presence and concentration of raspberry pomace, as well as the duration of maturation, play a crucial role in enhancing the functional quality of fermented dairy matrices.

### 3.26. Red Bayberry

Red bayberry (*Myrica rubra* Sieb. et Zucc.), commonly known as Chinese bayberry, is a fruit native to China and Southeast Asia. Due to the absence of a protective exocarp, this fruit exhibits poor storage stability and is therefore commonly processed into juice or wine, resulting in a significant amount of by-products [112]. Myrica rubra pomace, which constitutes approximately 20% of the fruit’s weight after juice extraction, is often discarded despite its richness in polyphenols and other bioactive compounds [113], indicating the need for further research to support its application in functional food development.

Zhu et al. [61] isolated two polysaccharide fractions (P1 and P2) from mixed fermented Chinese bayberry pomace wine and evaluated their antioxidant activity using DPPH, ABTS, and FRAP assays. Both fractions showed concentration-dependent increases in radical scavenging activity and reducing power, and their readdition to the wine affected phenolic content.

### 3.27. Soursop

Soursop (*Annona muricata* L.), widely cultivated in tropical regions, is valued for its distinctive flavor and purported health benefits. It is primarily consumed fresh or processed into a variety of commercial products, during which approximately 10–28.5% of the fruit is discarded as waste [114]. Soursop by-products, such as leaves, seeds, and peels, are rich in phenolic compounds with strong antioxidant activity, indicating the need for further research.

Bueno-Rojas et al. [39] evaluated the fermentation of kombucha using soursop leaves to valorize this underutilized by-product and assess its functional properties. Over a 10-day fermentation period, the total soluble phenol (TSP) content of the soursop leaf kombucha slightly decreased from 0.64 to 0.59 mg GAE/mL, with minor fluctuations and no consistent upward trend. Antioxidant capacity, as measured by the ABTS assay, remained constant at 0.13 mg TE/mL throughout fermentation, while DPPH radical scavenging activity showed a marginal increase from 0.10 to 0.12 mg TE/mL. The FRAP decreased from 0.85 to 0.75 mg TE/mL. These results suggest that while the soursop leaf kombucha maintained moderate antioxidant activity, fermentation did not markedly enhance its phenolic content or radical scavenging potential.

### 3.28. Strawberry

Strawberry (*Fragaria* × *ananassa* Duch.), one of the most popular fruits worldwide, is renowned not only for its sweet taste and vibrant red color. Due to its high perishability, most harvested strawberries are processed into various products, during which approximately 4–11% of the fruit mass remains as pomace by-product [115]. This processing residue, known as strawberry pomace, retains a considerable amount of phenolic compounds and antioxidant activity, making it a promising candidate for functional food applications. Therefore, further research is warranted to optimize its valorization through sustainable bioprocessing techniques.

Sady et al. [29] investigated the impact of microbial fermentation on the functional properties of strawberry pomace, utilizing three fermentation approaches: lactic acid bacteria (B), yeast (D), and a mixed culture of both (BD). Among the treatments, fermentation with yeast alone (D) resulted in the highest TPC, increasing from 67.56 to 78.72 mg GA/L. Antioxidant activity, as measured by ABTS, also showed an improvement under yeast fermentation, rising from 0.59 to 0.80 mmol Trolox/L. The DPPH assay revealed a comparable trend, with scavenging activity increasing from 0.14 to 0.21 mmol Trolox/L. Similarly, the FRAP value improved from 0.38 to 0.49 mmol FeSO_4_/L.

## 4. Food Applications of Fermented Fruit By-Products

For the application of fruit by-products in foods through fermentation, the production volume of these by-products is also an important factor. Given the wide diversity of fruits, both the composition and the yield of their by-products vary greatly. Such differences in by-product generation can serve as important indicators for their potential use in food applications, and the quantities of by-products from specific fruits are summarized in Table 2.

At the low end, some fruits generate only limited absolute amounts of by-products despite exhibiting extremely high residue fractions, for example, baru, which produces only about 2200 tons of by-products annually, even though most of the fruit mass becomes inedible during processing. In contrast, several major fruit species generate substantially larger quantities of recoverable biomass, such as mango with an estimated annual by-product production of approximately 22.5 million tons, banana with about 40 million tons per year, and orange with roughly 55 million tons generated annually. This quantitative gradient matters for application design because the absolute volume of available biomass, rather than the residue fraction alone, ultimately determines the technical feasibility, economic viability, and scalability of fermentation-based valorization and ingredient development. From a sustainability perspective, high-volume residues offer the greatest leverage, as even partial recovery can substantially reduce waste handling, disposal, and associated environmental burdens at regional scale. By contrast, low-volume residues with extreme residue fractions are more suitable for targeted, high-value applications, where environmental benefits arise primarily from ingredient substitution rather than bulk waste diversion. The table also records the reporting basis used by each source, such as fresh weight, dry matter, or processed mass, so comparisons should respect these units. Accordingly, fruits characterized by both high processing throughput and large annual by-product generation represent more realistic and sustainable substrates for industrial bioconversion, whereas low-volume residues are better suited for niche or high-value applications.

The practical utilization of fermented fruit by-products is broad, reflecting their versatility as functional ingredients. Applications range from alcoholic and non-alcoholic beverages to baked goods and dairy, as well as condiments and novel food ingredients.

**Figure 2:**
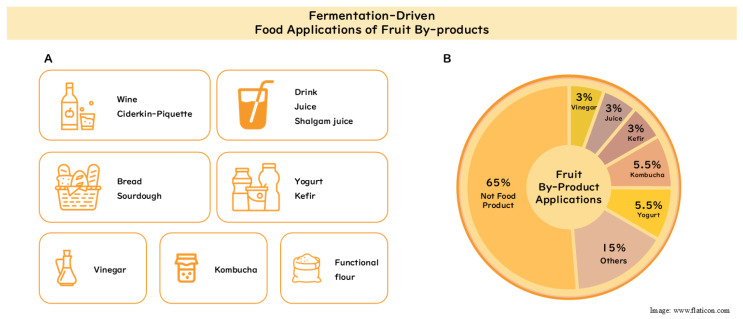
Overview of fermentation-driven food applications of fruit by-products. (**A**) Food application cases (Table 1 fermented product type); (**B**) proportion of studies with and without food applications.

Taken together, the studies mapped in Figure 2A show that fermented fruit by-products can function as ingredients across beverages, baked goods, cultured dairy, vinegar, kombucha, and functional flours, yet most reports remain at prototype scale and rely on in vitro antioxidant indices. Evidence on sensory acceptance, storage stability, safety, and nutrient bioaccessibility is limited. These gaps frame the conclusion’s call to move from extract-focused work to ingredient-level formulations validated in real processing and distribution conditions.

## 5. Limitations and Future Perspectives

Although this review compares a wide range of studies on fruit by-product fermentation, several constraints limit the feasibility of direct cross-study comparison. Fermentation conditions differ substantially in terms of time, temperature, moisture, inoculum level, strain identity, and process mode, and these variables can influence both the release and degradation of polyphenols [118]. At the same time, analytical practices vary in extraction procedures, calibration standards, and reporting bases, with results expressed on fresh weight, dry matter, or extract bases that are often not explicitly defined. Under these conditions, cross-study comparisons are inherently weak, and reported differences should be interpreted within the context of individual studies rather than as directly comparable outcomes. As a result, the identification of consistently superior substrates or fermentation strategies across studies remains unreliable under current reporting practices, highlighting the need for synthesis that emphasizes overall patterns and limitations rather than ranking individual results.

Common assays such as DPPH, ABTS, FRAP, and CUPRAC rely on different reaction mechanisms, and no single assay can therefore provide a complete or definitive measure of the antioxidant activity present in a sample, particularly in complex matrices such as fermented products. Fermentation alters pH, color, and turbidity and generates non-phenolic reducing substances, all of which can affect assay responses independently of polyphenol-related activity. For this reason, total polyphenol content is often measured to complement antioxidant capacity assays, although the Folin–Ciocalteu method is not phenolic-specific and shows limited sensitivity toward more lipophilic polyphenols, with results strongly influenced by extraction conditions [119]. Consequently, increases in total polyphenol content do not necessarily coincide with higher antioxidant capacity.

Claims regarding sustainability and industrial relevance are further constrained by insufficient definition of system boundaries. Valorization processes, including polyphenol recovery from by-products, inevitably introduce secondary environmental impacts through chemical and energy inputs, meaning that sustainability claims require boundary-defined assessment rather than being inferred from valorization alone [120]. Accordingly, future research should prioritize boundary-defined sustainability assessment that explicitly accounts for downstream processing, resource inputs, and waste generation when evaluating the industrial relevance of fermentation-based valorization.

## 6. Conclusions

Fermentation has been extensively investigated as an approach to enhance the antioxidant properties of fruit by-products; however, most studies remain focused on extract-level analysis. As summarized in Figure 2B, approximately 35% of the reviewed studies report the direct application of fermented fruit by-products in edible food or beverage products, indicating that food-formulated applications are less prevalent than extract-based approaches.

Across the studies reviewed, no single fruit by-product or fermentation strategy emerges as consistently superior. Reported changes in total polyphenol content and antioxidant activity vary with raw material, microbial strain, fermentation conditions, and analytical method, and this variability limits cross-study comparability and restricts the generalization of functional performance.

Fermented fruit by-products are therefore more commonly evaluated as sources of extractable compounds than as food ingredients. Although extraction remains useful for compositional characterization, many fermentation approaches associated with pronounced functional effects rely on long processing times, tightly controlled conditions, or specialized microbial strains. These features can hinder scalability and reduce compatibility with existing food manufacturing processes.

In summary, the primary challenge in fermentation-based valorization of fruit by-products lies in translating laboratory-scale observations into food-compatible and scalable processes. While extract-level characterization remains essential for understanding functional changes, future work should place greater emphasis on fermentation strategies that align with realistic processing conditions and food application requirements.

## Figures and Tables

**Table 1:** Fermentation of fruit by-products by source, product type, and microorganism with total phenolics and antioxidant outcomes.

Source Material	By-Product Source	Fermented Product Type	Microorganism	TPC	Antioxidant Activity of Fermented By-Products						Reference
					Free Radical Scavenging Activity				Reducing Power		
					ABTS	DPPH	HOSC	ORAC	CUPRAC	FRAP	
Acerola	Co-products (peel, seeds, and pomace)	Not Food Product	*Lactobacillus acidophilus*, *Lacticaseibacillus paracasei*	–	AL10 (0~48 h): 16.89~22.08 µmol/g ALA5 (0~48 h): 18.24~28.79 µmol/g	AL10 (0~48 h): 13.51~14.19 µmol/g ALA5 (0~48 h): 5.70~9.87 µmol/g	–	–	–	AL10 (0~48 h): 11.60~13.60 µmol FeSO_4_/g ALA5 (0~48 h): 19.70~28.40 µmol FeSO_4_/g	[16]
	By-products	Not Food Product	Spontaneous fermentation	0~120 h: 779.25~1628.30 mg EGA/100 g	0~120 h: 1.51~1.66 µmol TEAC/100 g	–	–	–	–	0~120 h: 361.17~428.53 µmol TEAC/100 g	[17]
Apple	Pomace	Ciderkin-piquette	*Saccharomyces cerevisiae*, *Saccharomyces paradoxus*	0.1 mg/mL	–	530.84 ± 2.17 TEAC µm/µL	–	–	–	1.02 ± 0.02 mM Ferrous Equivalent/µL	[18]
	Pomace	Not Food Product	*Lactobacillus rhamnos* *us*	Unfermented apple pomace: 383.00 μg/mL Day 3: 440.45 μg/mL Day 6: 480.80 μg/mL	–	Increasing 32% at least, in comparison with unfermented apple pomace	1.14-fold on average after fermentation	–	–	–	[19]
	Pomace	Not Food Product	*Lactiplantibacillus plantarum*, *Saccharomyces cerevisiae*, *Bacillus subtilis*	APM (Day 9): increased by up to 108.19% compared to unfermented materials	–	–	–	–	–	–	[20]
	Pomace	Not Food Product	*Actinomucor elegans*, *Umbelopsis isabellina*	*A. elegans*: ↑ 27% (Day 4), then ↓ *U. isabellina*: ↑ 12% (Day 6), then stable	–	*A. elegans*: ↑ 13.5% (Day 4), then ↓ *U. isabellina*: ↑ 3.5% (Day 6), then ↓ (All > 70%; strong activity)	–	–	–	–	[21]
	Pomace	Not Food Product	LAB strains, Spontaneous fermentation	*Lactiplantibacillus* spp.: 2.301~4.082 mg GAE/g dw AP FS: 3.035 mg GAE/g dw AP NF: 2.975 mg GAE/g dw AP	*Lactiplantibacillus* spp.: 62.78~82.61% FS: 73.45% NF: 75.40%	*Lactiplantibacillus* spp.: 71.88~95.02% FS: 76.43% NF: 96.04%	–	–	*Lactiplantibacillus* spp.: 670.04~1114.68 µM of Trolox equivalents FS: 879.00 µM of Trolox equivalents NF: 591.50 µM of Trolox equivalents	*Lactiplantibacillus* spp.: 742.76~1647.21 µM Fe^2+^/L_extract_ FS: 1168.12 µM Fe^2+^/L_extract_ NF: 1150.73 µM Fe^2+^/L_extract_	[22]
	Peel	Not Food Product	*Aspergillus oryzae*	Day 0~12: 137.35~314.71 GAE mg	Day 4~12: increased than day 0 (↓ IC_50_)	–	–	–	–	Day 4: proximate value to day 0 Day8, 12: increased than day 0	[23]
	Pomace	Kefir	Water kefir grains	WKGs-TAC and TLC: 3.72~245.54 mg GAE/g Extract WKB-TAC and TLC: 47.62~1404.76 mg GAE/g Extract	WKGs-TAC: 5.12~10.06 μmol TE/g Extract WKB-TAC: 5.84~199.76 μmol TE/g Extract	WKGs-TAC: 0.0005~0.0040 μmol TE/g Extract WKB-TAC: 0.0028~0.0507 μmol TE/g Extract	–	–	–	WKGs-TAC and TLC: 0.89~53.69 μmol TE/g Extract WKB-TAC and TLC: 8.26~351.01 μmol TE/g Extract	[24]
Araticum	Peel and Seeds	Not Food Product	*Lactobacillus acidophilus* spp., *Bifidobacterium animalis*	Unfermented: 55.55 ± 0.77 mg GAE/g Fermented mMRS-A *L. acidophilus* spp.: 579.46~614.81 µg EAG/mL Bb-12: 657.74 ± 16.81 µg EAG/mL NF: 537.37 ± 11.57 µg EAG/mL	Unfermented: 1019.20 ± 55.10 mM TE/g	Unfermented: 350.67 ± 6.14 mM ET/g Fermented mMRS-A *L. acidophilus* spp.: 1.69~1.90 mM ET/mL Bb-12: 2.05 ± 0.04 mM ET/mL NF: 1.82 ± 0.02 mM ET/mL	–	–	–	Unfermented: 1175.23 ± 25.65 mM ferrous sulfate/g Fermented mMRS-A after 48 h *L. acidophilus* spp.: 5.09~6.01 mM ferrous sulfate/mL Bb-12: 6.67 ± 0.16 mM ferrous sulfate/mL NF: 4.70 ± 0.48 mM ferrous sulfate/mL	[25]
Avocado	Leaf	Not Food Product	*Pediococcus acidilactici*, *Pediococcus pentosaceus*, *Levilactobacillus brevis*, *Lactiplantibacillus plantarum*, *Leuconostoc mesenteroides*	*L. mesenteroides* 215, 219T (24~96 h): 17.34~20.49 mg GAE/g dw *L. brevis* 4121T, 5354 (24~96 h): 17.83~29.39 mg GAE/g dw *L. plantarum* 748T, 9567 (24~96 h): 21.98~30.72 mg GAE/g dw *Pediococcus* spp. (24~96 h): 17.46~29.56 mg GAE/g dw	–	*L. mesenteroides* 215, 219T (24~96 h): 27.84~47.47 mg TE/g dw *L. brevis* 4121T, 5354 (24~96 h): 29.23~47.20 mg TE/g dw *L. plantarum* 748T, 9567 (24~96 h): 25.56~49.68 mg TE/g dw *Pediococcus* spp. (24~96 h): 28.90~51.32 mg TE/g dw	–	–	–	*L. mesenteroides* 215, 219T (24~96 h): 57.49~72.96 mg TE/g dw *L. brevis* 4121T, 5354 (24~96 h): 58.29~91.58 mg TE/g dw *L. plantarum* 748T, 9567 (24~96 h): 71.58~96.61 mg TE/g dw *Pediococcus* spp. (24~96 h): 50.34~93.33 mg TE/g dw	[26]
	Seed	Not Food Product	*Aspergillus oryzae*, *Aspergillus awamori*	*A. awamori* 0 h: 65.49 mg GAE/g AVS paste 96 h: 75.79 mg GAE/g AVS paste *A. oryzae* 48 h: 69.43 mg GAE/g AVS paste 72 h: 67.88 mg GAE/g AVS paste	–	Control 96 h: 32.63 umol TE/g AVS paste *A. oryzae* 96 h: 42.12 µmol TE/g AVS paste *A. awamori* 0 h: 51.17 µmol TE/g AVS paste 72 h: 66.57 µmol TE/g AVS paste 96 h: 75.82 µmol TE/g AVS paste	–	–	–	–	[27]
Banana	Peel	Vinegar	*Saccharomyces cerevisiae*, Acetic acid bacteria(Spontaneous fermentation)	4.03~5.72 mg GAE/L	–	5.42~60.92%	–	–	–	–	[28]
Baru	Mesocarp	Not Food Product	*Lactobacillus acidophilus* spp., *Bifidobacterium animalis*	Unfermented: 3.06 ± 0.33 mg GAE/g Fermented mMRS-B *L. acidophilus* spp.: 786.87~968.69 µg EAG/mL Bb-12: 982.15 ± 23.87 µg EAG/mL NF: 571.38 ± 25.42 µg EAG/mL	Unfermented: 167.66 ± 4.90 mM TE/g	Unfermented: 17.93 ± 0.26 mM ET/g Fermented mMRS-B *L. acidophilus* spp.: 3.45~4.14 mM ET/mL Bb-12: 4.11 ± 0.14 mM ET/mL NF: 2.70 ± 0.22 mM ET/mL	–	–	–	Unfermented: 110.58 ± 3.95 mM ferrous sulfate/g Fermented mMRS-B *L. acidophilus* spp.: 12.02~15.41 mM ferrous sulfate/mL Bb-12: 15.24 ± 0.20 mM ferrous sulfate/mL NF: 4.99 ± 0.62 mM ferrous sulfate/mL	[25]
Blackcurrant	Pomace	Not Food Product	*Lactobacillus acidophilus*, *Lactococcus lactis*, *Lactobacillus rhamnosus*, *Saccharomyces cerevisiae*	Control: 56.96 ± 2.11 mg GA/L B: 78.72 ± 1.28 mg GA/L D: 72.58 ± 1.28 mg GA/L BD: 70.91 ± 1.93 mg GA/L	Control: 0.57 ± 0.09 mmol Trolox/L B: 0.63 ± 0.10 mmol Trolox/L D: 0.58 ± 0.08 mmol Trolox/L BD: 0.55 ± 0.04 mmol Trolox/L	Control: 0.14 ± 0.04 mmol Trolox/L B: 0.36 ± 0.03 mmol Trolox/L D: 0.25 ± 0.05 mmol Trolox/L BD: 0.21 ± 0.04 mmol Trolox/L	–	–	–	Control: 0.39 ± 0.05 mmol FeSO_4_/L B: 0.73 ± 0.04 mmol FeSO_4_/L D: 0.40 ± 0.04 mmol FeSO_4_/L BD: 0.35 ± 0.03 mmol FeSO_4_/L	[29]
Chokeberry	Pomace	Not Food Product	*Lactobacillus acidophilus*, *Lactococcus lactis* subsp. *Lactis*, *Lactobacillus rhamnosus*, *Saccharomyces cerevisiae*	Control: 122.51 ± 0.84 mg GA/L B (LAB strains, Day 2): 90.43 ± 1.28 mg GA/L D (yeast strains, Day 2): 158.21 ± 3.17 mg GA/L BD (all the abovementioned strains, Day 2): 140.36 ± 2.69 mg GA/L	Control: 1.30 ± 0.09 mmol Trolox/L B: 1.08 ± 0.19 mmol Trolox/L D: 1.67 ± 0.07 mmol Trolox/L BD: 1.29 ± 0.03 mmol Trolox/L	Control: 0.44 ± 0.02 mmol Trolox/L B: 0.35 ± 0.04 mmol Trolox/L D: 0.67 ± 0.04 mmol Trolox/L BD: 0.59 ± 0.05 mmol Trolox/L	–	–	–	Control: 0.68 ± 0.09 mmol FeSO_4_/L B: 0.50 ± 0.03 mmol FeSO_4_/L D: 0.77 ± 0.05 mmol FeSO_4_/L BD: 0.75 ± 0.04 mmol FeSO_4_/L	[29]
	Pomace	Not Food Product	*Trichoderma viride*	Unfermented: 67.83 ± 1.04 mg·g^−1^ Fermented: 107.21 ± 0.95 mg·g^−1^	↑ 20.34% (initial ↓ then ↑)	Peek at day 6	–	Peek at day 6	–	–	[30]
Granadilla	Seeds	Not Food Product	*Aspergillus niger*	Initial moisture 50%, 48 h, 80% Acetone: 4713.3 mg GAE/100 g of GSF d.b. (highest data)	Initial moisture 50%, 168 h, 80% Acetone: 749.74 ± 2.65 µmol of Trolox/g of GSF d.b. (highest data)	Initial moisture 50%, 168 h, 80% Acetone: 214.99 µmol of Trolox/g of GSF d.b. (highest data)	–	–	–	Initial moisture 50%, 168 h, 80% Acetone: 708.02 ± 4.13 µmol of Trolox/g of GSF d.b. (highest data)	[31]
Grape	Pomace	Ciderkin-piquette	*Saccharomyces cerevisiae*, *Saccharomyces paradoxus*	≥0.55 mg/mL	–	705.79 ± 49.82 TEAC µm/µL	–	–	–	25.97 ± 0.11 mM Ferrous Equivalent/µL	[18]
	Pomace	Kombucha	SCOBY	20 g sucrose, 20 °C, Day 7: 507.14 ± 9.21 GAE mg/L (highest data)	–	20 g sucrose, 20 °C, Day 7: 1.08 ± 0.03 DPPH IC50 mL/L (highest data)	–	–	–	–	[32]
	Pomace Seeds	Not Food Product	*Aspergillus niger*, *Monascus anka*, *Eurotium cristatum*	*Eurotium cristatum*: 9.21-fold increase after 12 days (highest data)	*M. anka*: 3.64-fold increase after 12 days (highest data)	*Eurotium cristatum*: 3.91-fold increase after 12 days (highest data)	–	–	–	–	[33]
	Pomace	Not Food Product	*Aspergillus niger*, *Saccharomyces cerevisiae*, *Pichia stipitis*	–	–	Single culture: no increased Co-culture (120 h): 12.99 EqTrolox/L	–	–	–	Single culture: no increased Co-culture (120 h): 24.77 EqTrolox/L	[34]
	Pomace	Sourdough	*Lactiplantibacillus plantarum*	–	Unfermented: 0.11~0.62 mM Trolox eq Fermented: 0.18~1.02 mM Trolox eq	Unfermented: 7.0~74.1% Fermented: 13.1~95.2%	–	–	–	–	[35]
	Pomace	Not Food Product	*Trametes Versicolor*	Laboratory Jars: 76% decreased after 15 days Tray bioreactor: 77% decreased after 15 days	Laboratory Jars: 82% decreased after 15 days Tray bioreactor: 72% decreased after 15 days	Laboratory Jars: 82% decreased after 15 days Tray bioreactor: 83% decreased after 15 days	–	–	–	Laboratory Jars: 77% decreased after 15 days Tray bioreactor: 84% decreased after 15 days	[36]
	Pomace	Shalgam juice	*Saccharomyces cerevisiae*	Day 9~44: 799.23~1.102 mg GAE/L	Day 9~44: 4.52~8.63 mmol TE/L	Day 9~44: 2.83~3.68 mmol TE/L	–	–	–	–	[37]
Guava	Co-products(peel, seeds, and pomace)	Not Food Product	*Lactobacillus acidophilus*, *Lacticaseibacillus paracasei*	–	GL10 0~48 h: 18.50~21.05 µmol/g GLA5 0~48 h: 23.94~28.50 µmol/g	GL10 0~48 h: 9.14~10.77 µmol/g GLA5 0~48 h: 14.50~15.51 µmol/g	–	–	–	GL10 0~48 h: 15.30~20.70 µmol FeSO_4_/g GLA5 0~48 h: 19.80~28.40 µmol FeSO_4_/g	[16]
	By-products	Not Food Product	Spontaneous fermentation	0~120 h: 39.68~59.68 mg EGA/100 g	0~120 h: 0.70~1.17 µmol TEAC/100 g	–	–	–	–	0~120 h: 244.42~265.30 µmol TEAC/100 g	[17]
Jabuticaba	Peel	Bread	Yeast	–	–	–	–	Formulations: 123.0~929.8 µmol TE/g	–	–	[38]
Jackfruit	Leaf	Kombucha	SCOBY	Day 0~10: 0.23~0.43 mg GAE/mL	Day 0~10: 0.13 ± 0.00 mg TE/mL	Day 0~10: 0.13~0.14 mg TE/mL	–	–	–	Day 0~10: 0.41~0.59 mg TE/mL	[39]
	Seed	Drink	*Lactiplantibacillus plantarum*	Unfermented: 293.92 ± 2.09 mg GAE/g DM Fermented: 299.56 ± 1.98 mg GAE/g DM	–	Unfermented: 268.60 ± 10.80 mg AAE/g DM Fermented: 271.32 ± 6.57 mg AAE/g DM	–	–	–	–	[40]
Lemon	Peel	Juice	Yeast strains	Un-FLPJ: 422.62 ± 0.01 µg/g SA-FLPJ: 368.96 ± 0.33 µg/g SF-FLPJ: 1292.58 ± 1.07 µg/g	–	Un-FLPJ: 22,581.86 ± 0.4 EC50 ppm SA-FLPJ: 27,946.90 ± 1.96 EC50 ppm SF-FLPJ: 19,117.66 ± 2.41 EC50 ppm	–	–	–	–	[41]
Litchi	Seed	Vinegar	*Saccharomyces bayanus*, *Acetobacter pasteurianus*, *Acetobacter oryzoeni*	Acid hydrolysis: 431.77~881.36 mg GAE/L Enzymatic hydrolysis: 475.17~1001.88 mg GAE/L	–	Acid hydrolysis: 54.53~67.42% Enzymatic hydrolysis: 50.38~75.92%	–	–	–	–	[42]
Mandarin	Peel	Not Food Product	*Aspergillus niger*	UF-CRPP: 13.73 ± 0.74 mg GAE/g FI-CRPP: 15.81 ± 0.13 mg GAE/g FO-CRPP: 17.19 ± 0.02 mg GAE/g	UF-CRPP: 23.65 ± 0.82 μmol TE/g FI-CRPP: 29.07 ± 1.35 μmol TE/g FO-CRPP: 34.48 ± 0.17 μmol TE/g	UF-CRPP: 16.58 ± 0.26 μmol TE/g FI-CRPP: 17.91 ± 0.39 μmol TE/g FO-CRPP: 26.08 ± 0.26 μmol TE/g	–	–	–	–	[43]
Mango	Peel	Not Food Product	*Lactiplantibacillus plantarum*, *Bifidobacterium animalis*	Treatments: 2.106~3.276 mg GAE/100 mg DM	–	Treatments: 1.383~3.270 mM TE	–	–	–	Treatments: 0.454~1.220 mM TE	[44]
	Kernel	Functional Flour	*Aspergillus oryzae*, *Aspergillus awamori*, *Lactiplantibacillus plantarum*	MKF Control (0~96 h): 2.402~2.982 mg GAE/g *A.* spp. (48~96 h): 2.443~2.718 mg GAE/g MKWF Control (0~96 h): 0.913~1.526 mg GAE/g *A.* spp. (48~96 h): 3.095~7.949 mg GAE/g	MKF Control (0~96 h): 0.151~0.238 μM TE *A.* spp. (48~96 h): 0.150~0.470 μM TE MKWF Control (0~96 h): 41.414~49.119 μM TE *A.* spp. (48~96 h): 47.231~65.043 μM TE	MKF Control (0~96 h): 9.387~9.955 mM TE *A.* spp.(48~96 h): 7.972~14.822 mM TE MKWF Control (0~96 h): 14.663~16.869 mM TE *A.* spp. (48~96 h): 33.298~64.451 mM TE	–	–	–	–	[45]
Mulberry	Pomace	Not Food Product	*Lactobacillus plantarum*	–	Day 3: 480.22 ± 5.77 mg Trolox/100 mL (highest data)	Day 3: 275.06 ± 4.24 mg Trolox/100 mL (highest data)	–	Day 3: 331.05 ± 2.07 mg Trolox/100 mL (highest data)	–	Day 3: 334.19 ± 20.17 mg Trolox/100 mL	[46]
	Pomace	Yogurt	Starter culture	1% MPo (Day 1~28): 0.70~0.86 mg GAE/g 2% MPo (Day 1~28): 2.47~4.37 mg GAE/g 3% MPo (Day 1~28): 3.92~5.48 mg GAE/g	–	–	–	–	–	–	[47]
Orange	Peel	Not Food Product	*Penicillium camemberti*	Unfermented: 0.54 ± 0.05 mg GAE/g Fermented: 2.06 ± 0.55 mg GAE/g	–	Unfermented: 60.39 ± 1.40% Fermented: 81.68 ± 0.18%	–	–	–	Unfermented: 9.58 ± 0.42 mg AAE/100 g Fermented: 10.77 ± 0.27 mg AAE/100 g	[48]
	Peel	Not Food Product	*Levilactobacillus brevis*, *Lactiplantibacillus plantarum* strains	–	3 strains (24~48 h) and control: 9604.9~11,807.8 µg TE/g dw	3 strains (24~48 h) and control: 2214.4~2625.0 µg TE/g dw	–	–	–	–	[49]
	Peel	Not Food Product	*Trichoderma koningii*, *Aspergillus oryzae*, *Lactobacillus casei*	The optimal combination (strain ratio 1:5:7, inoculum amount 6%, fermentation temperature 30 °C): 515.37 μg GAE/mL	Significantly higher vs. Control	No significant difference vs. Control	–	–	–	Significantly higher vs. Control	[50]
	Pomace	Not Food Product	*Lactobacillus plantarum* strains, *Bacillus subtilis*	Unfermented: 5.55 mg GAE g^−1^ DW BPF increased the total phenolic content of Citrus Pomace by up to 133.15%	BPF: 24.83 Vc mg g^−1^ (increased 226.1%)	BPF: 18.76 Vc mg g^−1^ (increased 397.8%)	–	–	–	–	[51]
	Pomace	Juice	*Lactobacillus acidophilus*, *Lactobacillus casei*, *Lactobacillus plantarum*	Orange pomace samples (Day 0~4): 292.71~540.67 mg GAE/100 g DW	–	Orange pomace samples (Day 0~4): 231.40~300.98 mg TE/100 g DW	–	–	Orange pomace samples (Day 0~4): 235.06~423.01 mg TE/100 g DW	–	[52]
Pequi	Mesocarp and Exocarp	Not Food Product	*Lactobacillus acidophilus*, *Bifidobacterium animalis* subsp. *lactis*	*L. acidophilus* spp.: 1348.15~1398.65 µg EAG/mL Bb-12: 1472.73 ± 10.10 µg EAG/mL NF: 1425.59 ± 47.74 µg EAG/mL	–	*L. acidophilus* spp.: 11.78~12.17 mM ET/mL Bb-12: 11.62 ± 0.09 mM ET/mL NF: 13.4 ± 0.44 mM ET/mL	–	–	–	*L. acidophilus* spp.: 31.87~33.87 mM ferrous sulfate/mL Bb-12: 34.54 ± 1.00 mM ferrous sulfate/mL NF: 32.48 ± 1.51 mM ferrous sulfate/mL	[25]
Pineapple	Peel	Not Food Product	*Aspergillus niger* strains	–	*A. niger* HT3 (Treatment 7): 77.38 ± 6.64% *A. niger* Aa20 (Treatment 6): 81.41 ± 4.06%	*A. niger* HT3 (Treatment 6): 60.28 ± 1.74% *A. niger* Aa20 (Treatment 6, from F): 57.87 ± 0.46%	–	–	–	*A. niger* HT3 (Treatment 8): 176.64 ± 27.81 mEq Trolox/g *A. niger* Aa20 (Treatment 4): 113.39 ± 5.99 mEq Trolox/g	[53]
	Peel	Not Food Product	*Lactobacillus plantarum*, *Lactobacillus rhamnosus*, *Aspergillus oryzae*	*L. plantarum* (Day 5): ↑ 248.11% *A. oryzae* (Day 5): ↑ 182.0% *L. rhamnosus* (Day 5): ↑ 158.4%	–	*Lactobacillus* spp. Day 1~5: ↑ 48.52~238.52% (250~1000 µg/mL FPPE)	–	–	–	–	[7]
	Peel	Yogurt	*Lactobacillus delbrueckii*, *Streptococcus thermophilus*	Day 14: highest in UP	Day 14: highest in UP	Day 14: highest in UU	–	–	–	Day 14: highest in UU, 2.22 mmol/L	[54]
	Peel	Yogurt	*Lactobacillus bulgaricus*, *Streptococcus thermophilus*	Control: 52.207 µg/mL (lowest data) NPFU: 104.931 µg/mL (highest data)	↑ during storage; further ↑ with ultrasound	↑ during storage; further ↑ with ultrasound	–	–	–	Control: 1.833 mmol/L (lowest data) NPFU: 2.8667 mmol/L (highest data)	[55]
Pomegranate	Peel	Not Food Product	*Aspergillus tubingensis*	Control, 48 h: 167.7 mg GAE/g DM 1:10 ratio, 48 h: 300.3 mg GAE/g DM (highest data)	–	Control, 48 h: 156.44 mg GAE/g DM 1:10 ratio, 48 h: 256.34 mg GAE/g DM (highest data)	–	–	–	–	[56]
Pomelo	Peel	Not Food Product	*Aspergillus oryzae*	Day 0~12: 19.46~74.67 mg GAE/g	Day 0~12: 4.09~35.49 mg VCE/g	Day 0~12: 2.37~3.84 mg VCE/g	Day 0~12: 88.74~103.88 mg VCE/g	–	–	Day 0~12: 2.06~7.03 mg TE/g	[57]
Rambutan	Peel	Not Food Product	*Saccharomyces cerevisiae*, *Yarrowia lipolytica*	Treatment 1~15: 55.33~103.66 mg/g	Treatment 1~15: 94.00~100.00%	Treatment 1~15: 47.2~63%	–	–	–	–	[58]
	Peel	Not Food Product	*Aspergillus niger*	Treatment 10: 73.18 ± 0.29 mg/g (highest data)	Treatment 10: 98.44 ± 0.14% (highest data)	Treatment 10: 74.73 ± 0.11% (highest data)	–	–	–	–	[59]
Raspberry	Pomace	Not Food Product	*Lactobacillus acidophilus*, *Lactococcus lactis*, *Lactobacillus rhamnosus*, *Saccharomyces cerevisiae* strains	Control: 80.39 ± 1.74 mg GA/L B: 69.79 ± 3.02 mg GA/L D: 82.06 ± 2.94 mg GA/L BD: 77.32 ± 1.67 mg GA/L	Control: 0.42 ± 0.05 mmol Trolox/L B: 0.33 ± 0.06 mmol Trolox/L D: 0.70 ± 0.16 mmol Trolox/L BD: 0.41 ± 0.17 mmol Trolox/L	Control: 0.20 ± 0.05 mmol Trolox/L B: 0.21 ± 0.04 mmol Trolox/L D: 0.36 ± 0.13 mmol Trolox/L BD: 0.17 ± 0.02 mmol Trolox/L	–	–	–	Control: 0.27 ± 0.05 mmol FeSO_4_/L B: 0.29 ± 0.07 mmol FeSO_4_/L D: 0.47 ± 0.02 mmol FeSO_4_/L BD: 0.25 ± 0.03 mmol FeSO_4_/L	[29]
	Pomace	Kefir	Kefir starter	K2: 78.24 ± 3.29 mg/L (9-fold higher than control)	–	K1~K4: 15.5~95.91%	–	–	–	–	[60]
Red Bayberry	Pomace	Wine	Yeast, lactic acid bacteria, and acetic acid bacteria	–	Concentration-dependent increase	Concentration-dependent increase	–	–	–	Concentration-dependent increase	[61]
Soursop	Leaf	Kobucha	SCOBY	Day 0~10: 0.59~0.74 mg GAE/mL	Day 0~10: 0.12~0.13 mg TE/mL	Day 0~10: 0.08~0.12 mg TE/mL	–	–	–	Day 0~10: 0.56~0.89 mg TE/mL	[39]
Strawberry	Pomace	Not Food Product	*Lactobacillus acidophilus*, *Lactococcus lactis* subsp. *Lactis*, *Lactobacillus rhamnosus*, *Saccharomyces cerevisiae*	Control: 67.56 ± 1.28 mg GA/L B: 71.46 ± 1.67 mg GA/L D: 78.72 ± 2.94 mg GA/L BD: 77.04 ± 3.77 mg GA/L	Control: 0.59 ± 0.09 mmol Trolox/L B: 0.71 ± 0.07 mmol Trolox/L D: 0.80 ± 0.03 mmol Trolox/L BD: 0.76 ± 0.06 mmol Trolox/L	Control: 0.14 ± 0.09 mmol Trolox/L B: 0.15 ± 0.07 mmol Trolox/L D: 0.21 ± 0.04 mmol Trolox/L BD: 0.20 ± 0.02 mmol Trolox/L	–	–	–	Control: 0.38 ± 0.04 mmol FeSO_4_/L B: 0.35 ± 0.04 mmol FeSO_4_/L D: 0.49 ± 0.07 mmol FeSO_4_/L BD: 0.38 ± 0.02 mmol FeSO_4_/L	[29]

**Table 2:** Proportion and Annual Production of Fruit By-Products.

Fruit	By-Product Type	Proportion	Basis	Annual By-Product Production	Reference
Acerola	Seed, bagasse, ripe fruit, peel, and pulp	40%	Fruit volume	24,400 tons (Brazil)	[63]
Apple	Pomace	25–30%	Residue	5–7 million tons	[64]
Araticum	Peel and seed	45–55%	Fruit’s mass	–	[68]
Avocado	Peel and seed	20–30%	Fruit	1.2 million tons	[70]
Banana	Peel	35%	Total fruit weight	40 million tons	[72]
Baru	Epicarp, mesocarp, and endocarp	95.7%	Processed fruit mass	2200 tons	[76,116]
Blackcurrant	Pomace	25–30%	Fruit weight	15,000–20,000 tons (Poland)	[78]
Chokeberry	Pomace	16–30%	*w*/*w* of fruit	10,000 tons (Poland)	[80]
Granadilla	Peel	50–60%	Total fruit weight	1 million tons (China)	[82]
Grape	Pomace	16.7%	*w*/*v* of fruit	10.5–13.1 million tons	[84]
Guava	Residue	30%	Fruit weight	2.25 million tons	[1,87]
Jabuticaba	Peel, seed, and adhered pulp	40%	Whole fruit	–	[88]
Jackfruit	Peel and seed	80%	Fruit weight	2.96 million tons	[89]
Lemon	Peel, seed, and pulp	50%	Fruit	–	[92]
Litchi	Peel and seed	30–40%	Mass of fruit	0.54 million tons	[93]
Mandarin	Peel	30%	Wet fruit mass	60,000 tons (Korea)	[96]
Mango	Peel and seed	30–60%	Fruit weight	20 million tons	[5]
Mulberry	Pomace	40%	Total fruit weight	–	[101]
Orange	Peel, seed, and pulp	60%	Fresh weight	55 million tons	[6]
Pequi	Endocarp, seed, and peel	91%	Fruit mass	72,000 tons	[104]
Pineapple	Crown, peel, and core	80%	Total fruit weight	22.5 million tons	[7]
Pomegranate	Rind and seed	54%	Fruit	1.62 million tons	[107]
Pomelo	Peel	30–50%	*w*/*w* of fruit	2.8–4.7 million tons	[108]
Rambutan	Peel, seed, and embryo	61.3%	Dry weight of fruit	–	[109]
Raspberry	Pomace	9%	Weight of processed fruit	0.5 million tons	[111,117]
Red Bayberry	Pomace	20%	Fruit’s weight	–	[113]
Soursop	Peel and seed	10–28.5%	Fruit	–	[114]
Strawberry	Pomace	4–11%	Fruit weight	–	[115]

## Data Availability

No new data were created or analyzed in this study. Data sharing is not applicable to this article.

## Abbreviations

AL10	Acerola co-product + *L. paracasei* L-10
ALA5	Acerola co-product + *L. acidophilus* LA-05
AP	Apple pomace
APM	75% apple pomace and 20% corn bran
B	Bacteria
Bb-12	*Bifidobacterium animalis* subsp. *Lactis* Bb-12
BD	Bacteria and yeast
D	yeast
d b	Dry basis
DM	Dry matter
EGA	equivalent of gallic acid
FPPE	Fermented pineapple peel extracts
GAE	Gallic acid equivalent
GL10	Guava + *Lacticaseibacillus paracasei* L-10
GLA5	Guava + *Lactobacillus acidophilus* LA-05
GSF	Granadilla seed flour
IC50	Half maximal inhibitory concentration
K1	Kefir with 10% raspberry pomace
K2	Kefir with 20% raspberry pomace
K4	Kefir with 40% raspberry pomace
MKF	Mango kernel flour
MKWF	Mango kernel water-soluble fraction
mMRS-A	Modified MRS broth with araticum
mMRS-B	Modified MRS broth with baru
mMRS-P	Modified MRS broth with pequi
MPo	Mulberry pomace
NF	Non-fermented
NPFU	Contained 5% (*w*/*v*) pineapple dietary fiber, for which the preheated milk was inoculated by the strains, followed by the ultrasound pretreatment
SA-FLPJ	Sugar-added fermented lemon peel juice
SD	Sourdough
SF-FLPJ	Sugar-free fermented lemon peel juice
TE	Trolox equivalent
TEAC	Trolox equivalent antioxidant capacity
Un-FLPJ	Unfermented lemon peel juice
UP	Ultrasonically stressed inocula combined with the addition of conventional extraction solution
UU	Ultrasonically stressed inocula combined with the addition of ultrasonication-assisted peel extract
VCE	Vitamin C equivalent
WKGs-TAC	Amphiphilic lipids extracted from water kefir grains
WKGs-TLC	Lipophilic lipids extracted from water kefir grains
WKB-TAC	Amphiphilic lipids extracted from water kefir beverage
WKB-TLC	Lipophilic lipids extracted from water kefir beverage
